# Role of receptor polymorphism and glycosylation in syncytium induction and host range variation of ecotropic mouse gammaretroviruses

**DOI:** 10.1186/1742-4690-5-2

**Published:** 2008-01-10

**Authors:** Yuhe Yan, Yong T Jung, Tiyun Wu, Christine A Kozak

**Affiliations:** 1 Laboratory of Molecular Microbiology, National Institute of Allergy and Infectious Diseases, Bethesda, MD, 20892-0460, USA; 2 Department of Microbiology, Dankook University, Cheonan, 330-714, Korea; 3 Laboratory of Molecular Genetics, National Institute of Child Health and Development, Bethesda, MD, 20892, USA

## Abstract

**Background:**

We previously identified unusual variants of Moloney and Friend ecotropic mouse gammaretroviruses that have altered host range and are cytopathic in cells of the wild mouse species *Mus dunni*. Cytopathicity was attributed to different amino acid substitutions at the same critical *env *residue involved in receptor interaction: S82F in the Moloney variant Spl574, and S84A in the Friend mouse leukemia virus F-S MLV. Because *M. dunni *cells carry a variant CAT-1 cell surface virus receptor (dCAT-1), we examined the role of this receptor variant in cytopathicity and host range.

**Results:**

We expressed dCAT-1 or mCAT-1 of NIH 3T3 origin in cells that are not normally infectible with ecotropic MLVs and evaluated the transfectants for susceptibility to virus infection and to virus-induced syncytium formation. The dCAT-1 transfectants, but not the mCAT-1 transfectants, were susceptible to virus-induced cytopathicity, and this cytopathic response was accompanied by the accumulation of unintegrated viral DNA. The dCAT-1 transfectants, however, did not also reproduce the relative resistance of *M. dunni *cells to Moloney MLV, and the mCAT-1 transfectants did not show the relative resistance of NIH 3T3 cells to Spl574. Western analysis, use of glycosylation inhibitors and mutagenesis to remove receptor glycosylation sites identified a possible role for cell-specific glycosylation in the modulation of virus entry.

**Conclusion:**

Virus entry and virus-induced syncytium formation using the CAT-1 receptor are mediated by a small number of critical amino acid residues in receptor and virus Env. Virus entry is modulated by glycosylation of cellular proteins, and this effect is cell and virus-specific.

## Background

The CAT-1 receptor mediates the entry of ecotropic gammaretroviruses into rodent cells. Virus properties that rely on receptor recognition such as host range or pathogenicity could potentially be affected by polymorphisms that alter the receptor or the receptor binding domain (RBD) of the virus. In previous studies we identified two unusual ecotropic mouse leukemia virus (MLV) variants [1,2]. Both of these viruses have altered host range, both are cytopathic, and both have amino acid substitutions at the same site in their RBDs. Spl574 is a Moloney MLV (MoMLV) variant with the substitution S82F, and F-S MLV is a Friend MLV (FrMLV) variant with the substitution S84A. Both viruses cause the formation of large multinucleated syncytia on cells derived from the wild mouse species *M. dunni *two days after infection, and syncytium formation is accompanied by the accumulation of large amounts of unintegrated viral DNA [2]. These two viruses also differ from each other and from their respective parental MLVs in host range. Spl574 replicates efficiently only in *M. dunni *cells and very inefficiently in other mouse cells such as NIH 3T3 and SC-1 cells. F-S MLV shows no unusual pattern of infectivity in mouse cells, but is capable of infecting hamster cells that are normally resistant to ecotropic MLVs.

**Figure 1:**
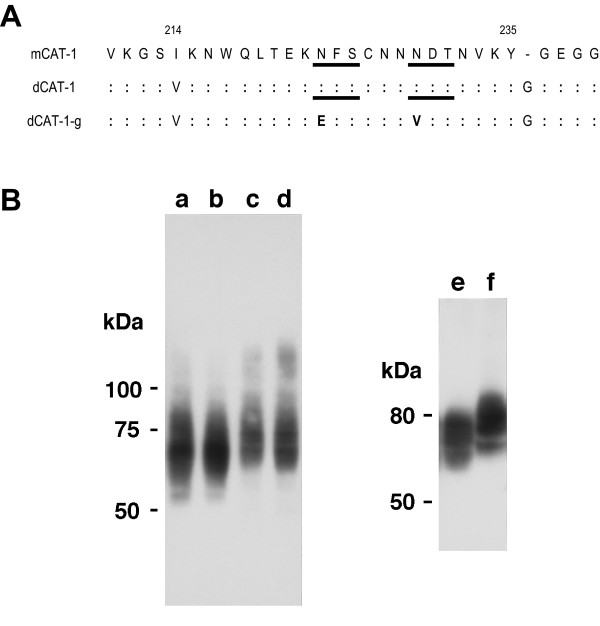
**(A) **Comparison of the deduced amino acid sequences of the third extracellullar loop of the CAT-1 receptor. Glycosylation sites are underlined. Sequences for mCAT-1 (NIH 3T3) and dCAT-1 (*M. dunni*) were previously determined (GenBank accession no. M26687, [6]). dCAT-1-g was generated by mutagenesis. **(B) **Expression of HA-tagged CAT-1 genes in various cell lines. a, Tb-1-Lu cells with mCAT-1; b, Tb-1-Lu with dCAT-1; c, MA139 cells with mCAT-1, d and e, MA139 with dCAT-1; f, *M. dunni *with m-CAT-1. Cell lysates were electrophoresed on 8% (right panel) or 10% gels. Molecular weight markers are given for each panel.

The fact that these two viruses are only cytopathic in *M. dunni *cells suggests involvement of the receptor-virus interaction for two reasons. First, the amino acid residue that is modified in both viruses has been identified as one of the critical amino acids forming the receptor binding site [3,4]. Second, *M. dunni *cells differ from other mouse cells in their resistance to MoMLV [5], and these cells are known to carry a modified CAT-1 receptor (dCAT-1). The dCAT-1 gene of *M. dunni *cells differs from the prototypical CAT-1 gene of the laboratory mouse (mCAT-1) in that the third extracellular loop that contains the virus binding region has a substitution (I214V) as well as an inserted glycine after Y235, a residue critical for receptor function [6] (Fig. 1A).

In this study, we examined the role of the dCAT-1 receptor in syncytium formation and susceptibility to infection by different ecotropic MLVs. We generated an expression vector containing dCAT-1 and transfected either this clone or the mCAT-1 gene into cells of non-rodent species that are not normally infectible by ecotropic virus. The transfected cells were then evaluated for susceptibility to infection by ecotropic MLVs and for virus induced syncytia. While virus induced syncytia were only seen in the dCAT-1 transfectants, a different panel of virus isolates was capable of efficiently infecting and/or inducing syncytia in these transfectants suggesting that virus-cell fusion and cell-cell fusion are distinct receptor mediated phenomena. The possible contribution of differential glycosylation to these phenotypic differences was evaluated using Western analysis, treatment by glycosylation inhibitors and mutagenesis to remove glycosylation sites.

## Results

### Syncytium formation in cells expressing mCAT-1 or dCAT-1

HA-tagged mCAT-1 and dCAT-1 clones were transfected into three cell lines that are not naturally susceptible to infection by ecotropic mouse gammaretroviruses: MA139 (ferret), Tb-1-Lu (bat lung), and MDCK (canine kidney) cells. As a control, mCAT-1 was transfected into *M. dunni *cells. Pools of stably transfected cells were used for analysis along with single cell derived clones of transfected MA139 cells. mCAT-1 and dCAT-1 expression in transfected cells was confirmed by Western analysis (Fig. 1B). Consistent with previous observations [7], CAT-1 was detected as a heterogeneously glycosylated protein in each cell line. The size range distribution for the mCAT-1 and dCAT-1 proteins was similar for each cell line, but the size range and band patterns were variable between cell lines suggesting cell specific differences in glycosylation. Thus, for example, the molecular weight range of CAT-1 was lower in MDCK cells (not shown) and Tb-1-Lu cells than in MA139 cells and *M. dunni *cells (Fig. 1B).

**Figure 2:**
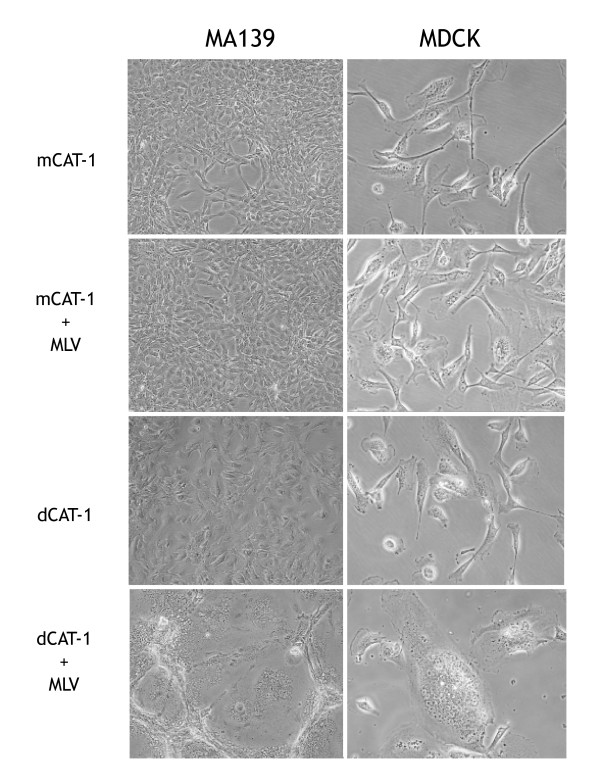
Cytopathic effects of virus infection on two cell lines, MA139 and MDCK cells, transfected with mCAT-1 or dCAT-1. Transfected MA139 cells are shown in the four panels on the left, transfected MDCK cells on the right. To the left are indicated which transfected CAT-1 gene is expressed and which cells are MLV infected. Cultures were photographed 3 days after one of two plates of each transfected cell was infected with F-S MLV. Panels show representative fields of cultures infected with undiluted virus stock. Objective lens magnification was 20× for the MA139 cells and 40× for the MDCK cells.

These stable transfectants of MA139, Tb-1-Lu and MDCK cells were infected with a panel of ecotropic gammaretroviruses including two, Spl574 and F-S MLV that induce multinucleated syncytia in *M. dunni *cells but not in other mouse cell lines. The infected cells were examined for cytopathicity over a period of 2–5 days. Transfectants of all 3 cell lines expressing mCAT-1 showed no signs of cytopathicity following virus infection as shown for the MA139 and MDCK transfectants in Fig. 2. In contrast, dCAT-1 expressing MA139 and MDCK cells (Fig. 2) as well as Tb-1-Lu cells (not shown) formed syncytia within two days of infection with Friend virus isolate F-S MLV.

Several separate pools of MA139 transfected cells were generated and tested. Virus-induced syncytia were observed in two independently derived pools of dCAT-1 transfected MA139 cells as well as three independently isolated clonal lines (FerrD2, N65FerrC2 and N65FerrB6), but not in 3 independently derived pools of mCAT-1 transfected MA139 cells.

Among the ecotropic isolates tested, F-S MLV was most efficient in inducing syncytia in all dCAT-1 transfected cells (Fig. 2), but syncytium formation was also observed following infection with the Friend MLV isolates FBLV and FrMLV57. Infection with MoMLV or Spl574 occasionally resulted in syncytium formation in these transfectants, but these syncytia were smaller and fewer in number, and, appeared 1–2 days after the appearance of syncytia in parallel cultures infected with the most cytopathic isolate, F-S MLV. Thus, cells expressing the dCAT-1 receptor can, like *M. dunni*, produce syncytia in response to virus infection, but these transfectants differ from *M. dunni *in their relative insensitivity to Spl574 and their sensitivity to syncytium formation by virus isolates that are not typically cytopathic in *M. dunni *cells.

### Accumulation of unintegrated viral DNA in infected transfected cells

**Figure 3:**
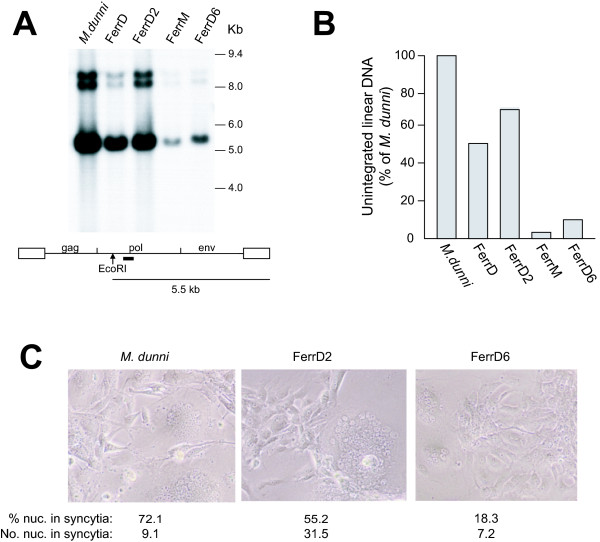
Unintegrated viral DNA in F-S MLV infected *M. dunni *cells and MA139 cells transfected with mCAT-1 or dCAT-1. Transfectant FerrM expresses mCAT-1 and the various FerrD transfectants express dCAT-1. **(A) **Southern blot of DNAs extracted by the Hirt method [25] three days after infection with F-S MLV. DNAs were cleaved with EcoRI and hybridized with a probe representing the segment of *pol *indicated by the black bar. **(B) **The 5.5 kb bands representing the *pol*-probe reactive cleavage product of linear viral DNA were quantified by densitometric scanning and compared to that of *M. dunni*, which was defined as 100%. **(C) **The indicated cultures of F-S MLV infected cells were photographed just before Hirt DNA extraction using objective lens magnification of 10×. The extent of virus induced fusion in each culture is indicated by the number of nuclei per cell and the proportion of nuclei in syncytia; numbers represent averages for 4–6 representative fields.

Virus induced syncytium formation in *M. dunni *cells was previously shown to be accompanied by the appearance of high levels of unintegrated viral DNA [2], a phenomenon also observed for other pathogenic retroviruses [8]. To determine if the transfected cells show this same response to cytopathic virus, we extracted Hirt DNA from CAT-1 transfected MA139 cells 3 days after infection with F-S MLV (Fig. 3A).

At the time of DNA extraction, FerrM cells, expressing mCAT-1, showed no cytopathic response and the observed level of unintegrated viral DNA was low (4.0% of *M. dunni*, Fig. 3A,B). In contrast, virus-induced syncytia were observed in all 3 dCAT-1 transfectants. 2 of these 3 transfectants had large multinucleated syncytia that involved >50% of the cells in infected cultures (Fig. 3C); levels of unintegrated linear DNA in these cells were high (53% and 68% of *M. dunni*) (Fig. 3A,B). The third dCAT-1 transfectant showed fewer and smaller syncytia (Fig. 3C); increased unintegrated DNA was detected in this line but levels were only about twice that of FerrM (Fig. 3A,B), Thus, viral DNA accumulation is observed in dCAT-1 but not mCAT-1 transfectants, increased viral DNA is associated with virus-induced cytopathicity in the transfected cells, and the amount of viral DNA varies with the severity of the cytopathic response.

### Virus replication in cells expressing CAT-1

To define the relationship between syncytium formation and productive virus infection, transfected cell lines carrying either mCAT-1 or dCAT-1 were tested for susceptibility to a panel of ecotropic MLVs using the XC plaque overlay test (Table 1). In this assay, clusters of infected cells expressing ecotropic Env glycoprotein are identified by plaques of syncytia formed by overlaid rat XC cells [9]. For the cytopathic viruses Spl574 and F-S MLV, the number of syncytia induced directly by these viruses in susceptible cells is approximately equivalent to the titer determined by this XC overlay assay; for example, parallel cultures of infected *M. dunni *cells produced an XC titer of 10^5.1 ^(Table 1) compared to Spl574 syncytium titer of 10^4.6^.

**Table 1:** Virus titers of ecotropic gammaretroviruses on mouse cells and mouse or ferret MA139 cells transfected with mCAT-1 or dCAT-1

	**Log_10 _Virus Titer^a^**			
	
**Cells^b^**	**F-S MLV**	**FBLV**	**Spl574**	**MoMLV**
NIH 3T3	5.7	5.3	**3.2**	5.1
*M. dunni*	5.1^c^	4.1	5.1^c^	**2.2**
*M. dunni *(mCAT-1)	5.5^c^	5.5	5.2^c^	4.5
FerrM (mCAT-1)	4.5	4.2	**0.3**	3.9
FerrD2 (dCAT-1)	4.7^c^	4.2^c^	**0.8**	4.2

^a^Virus titers were determined by the XC overlay test in which the indicated cells were infected with virus dilutions, irradiated and overlaid with XC cells to identify virus infected cells [9]. Titers represent the number of XC PFU in 0.2 ml. Infections were done four times; results shown are from one representative experiment. **Bold** figures represent titers reduced by more than 95% (1.3 log_10_) relative to NIH 3T3 (for MoMLV) or *M. dunni *cells (for Spl574).

^b^CAT-1 gene expressed in transfected cells is given in ().

^c^Syncytia were observed 2 days after virus infection.

Transfected *M. dunni *cells expressing mCAT-1 in addition to the endogenous dCAT-1 gene were significantly more susceptible to MoMLV infection than untransfected *M. dunni *cells (Table 1), consistent with a previous study indicating that the dCAT-1 sequence variation is responsible for *M. dunni *resistance to MoMLV [6]. No difference was noted in the XC plaque titer of Spl574 in *M. dunni *cells expressing mCAT-1 in addition to the endogenous dCAT-1, and no viruses other than Spl574 and F-S MLV were cytopathic in the mCAT-1 transfected *M. dunni *cells.

The differences between *M. dunni *and NIH 3T3 cells in susceptibility to ecotropic viruses were not reproduced in MA139 cells expressing dCAT-1 (FerrD2) or mCAT-1 (FerrM). In fact, there were no significant differences in the XC titers of different MLVs in FerrD2 and FerrM (Table 1). FBLV, F-S MLV, and, surprisingly, MoMLV efficiently infected both FerrM and FerrD2 with slightly higher XC titers for all viruses in FerrD2. Also, even though Spl574 efficiently replicates in *M. dunni*, Spl574 produced comparably low XC titers in both FerrD2 and FerrM. Thus, FerrD2 does not resemble *M. dunni *cells in its susceptibility to infection by MoMLV and Spl574; this difference suggests the involvement of additional factors independent of the CAT-1 receptor sequence.

The cytopathicity of different virus isolates did not always correlate with the efficiency of virus replication in FerrD2 as determined by XC virus titer. While on the one hand, Spl574 produced low XC titers on FerrD2 (Table 1) and was also poorly cytopathic, high XC titer viruses did not all produce syncytia in these cells. Thus, the most cytopathic virus in FerrD2 cells, F-S MLV, produced an XC titer comparable to that of the rarely cytopathic MoMLV. Efficient virus replication is thus not sufficient to generate a cytopathic response.

### Pseudotype infections

To further investigate the observed differences in XC titers for cells expressing different CAT-1 genes, we assessed infectivity using viral pseudotypes in a single round infectivity assay (Table 2). We infected mouse cells and transfected MA139 cells with the pCLMFG-LacZ vector pseudotyped with the envelopes of FrMLV57, MoMLV, and Spl574.

**Table 2:** Titers of ecotropic LacZ pseudotypes in mouse cells and MA139 cells transfected with mCAT-1 (FerrM) or dCAT-1 (FerrD2)

	**Log_10 _Titer of LacZ Pseudotypes^a^**		
	
**Cells^b^**	**FrMLV**	**Spl574**	**MoMLV**
NIH 3T3	3.8	**2.7**	4.0
*M. dunni*	2.7	4.5	**---**
FerrM (mCAT-1)	3.8	4.4	4.1
FerrD2 (dCAT-1)	4.2	4.9	4.2

^a^Measured as the number of cells positive for β-galactosidase activity in 100 ul. ---, no positive cells in cultures infected with 0.1 ml of undiluted pseudotype stock. **Bold** figures represent titer reduced by more than 95% (1.3 log_10_) relative to susceptible NIH 3T3 or *M. dunni *cells.

^b^CAT-1 gene expressed in transfected cells is given in ().

Infection with the MoMLV pseudotype is restricted in *M. dunni *cells (Table 2) as observed previously [1]. However, both of the transfected MA139 lines, FerrD2 and FerrM, were equally susceptible to the MoMLV pseudotype; the LacZ titers for this pseudotype in the two MA139 transfectants were similar to that of fully susceptible NIH 3T3 cells. This result is consistent with the XC test results showing high XC titers for MoMLV in both of these transfectants (Table 1).

The Spl574 pseudotype is restricted in NIH 3T3 cells as is the Spl574 virus (Tables 1, 2) suggesting that this restriction is entry related. In contrast, the Spl574 pseudotype was not restricted in FerrD2 or FerrM cells although Spl574 virus produces low XC titers in both of these transfectants. This shows that the failure of Spl574 to replicate efficiently in the transfected cells is not entry related and suggests the involvement of factor(s) restricting post-entry stages of Spl574 virus replication in ferret cells.

### Syncytium formation and virus replication in cells expressing dCAT-1 lacking glycosylation sites

*M. dunni *and FerrD2 cells express the same dCAT-1 receptor, but these cells differ in their relative infectivity by MoMLV and Spl574, and they produce syncytia in response to different virus isolates. One possible explanation for these differences is that CAT-1 may undergo different post-translational modification in the two cell lines. It has been shown that resistance of *M. dunni *cells to MoMLV infection is reduced by treatment with the inhibitor of glycosylation, tunicamycin (Tu) [10]. The involvement of glycosylation is also suggested by the observation that the CAT-1 glycosylation patterns differ in transfected MA139 and *M. dunni *cells (Fig. 1B; lanes e,f). To determine if glycosylation contributes to the observed differences, we generated a dCAT-1 clone from which the N-glycosylation sites had been removed.

**Figure 4:**
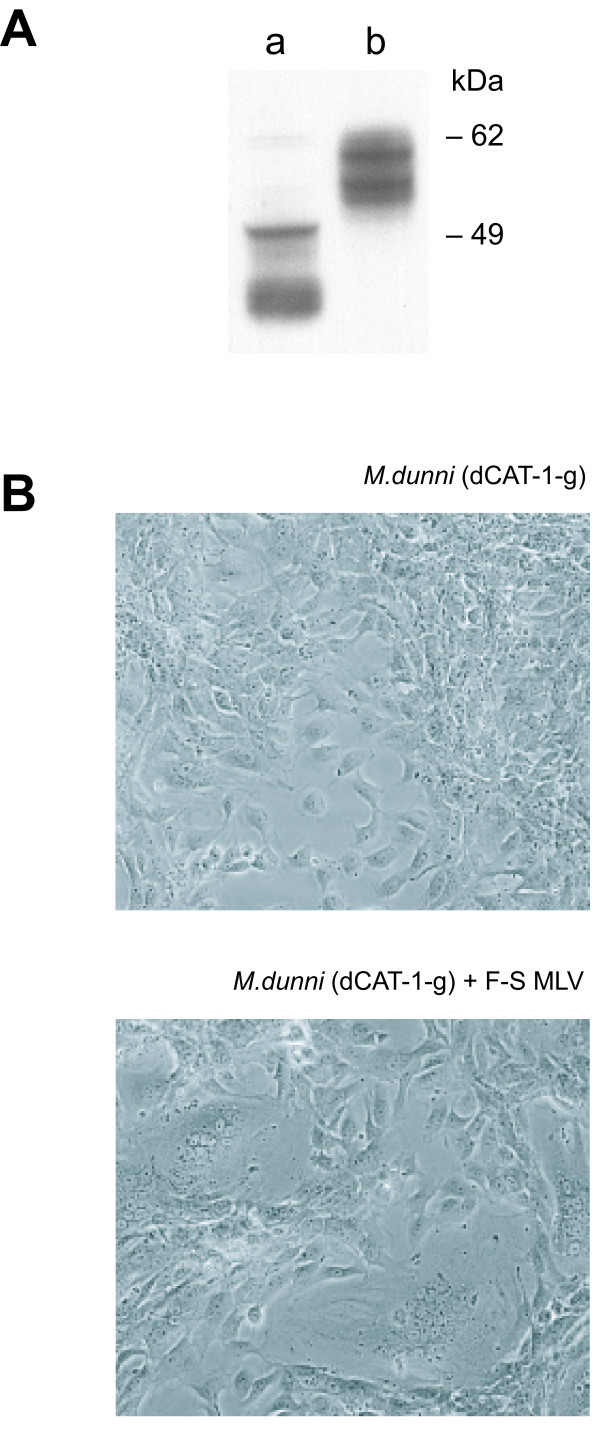
**(A) **Expression of HA-tagged dCAT-1 (lane b) or dCAT-1-g (lane a) in transfected *M. dunni *cells. The deglycosylated dCAT-1-g protein is approximately 55 kDa. This lane contains a low molecular weight smear likely to be HA-containing breakdown artifacts; this smear is seen to varying degrees in all transfectants expressing this construct. **(B) **Syncytium formation in MA139 cells expressing dCAT-1-g. Cells were photographed two days after cells in the bottom panel were infected with F-S MLV.

The CAT-1 protein has two glycosylation sites, and both carry N-glycans [7]. Both sites are in the third extracellular loop which also contains the residues implicated in virus binding and entry [11,12]. Both glycosylation sites were removed by PCR mediated site-specific mutagenesis from the dCAT-1 variant (Fig. 1), and the resulting clone, dCAT-1-g, was transfected into MA139 and *M. dunni *cells. Western analysis confirmed the presence of a single band of about 55 kDa as shown for transfected *M. dunni *cells in Fig. 4A. Attempts to generate stable *M. dunni *transfectants overexpressing dCAT-1 were not successful.

Expression of dCAT-1-g in *M. dunni *cells did not alter susceptibility to virus-induced syncytium by Spl574 or F-S MLV, nor did it result in syncytium formation by viruses not cytopathic in *M. dunni *cells. The transfectants, however, showed increased susceptibility to MoMLV compared to untransfected *M. dunni *cells (Table 3, Exp.1), as also shown above for *M. dunni*(mCAT-1) (Table 1); the transfectants showed no increase in their susceptibility to other ecotropic viruses. This is consistent with the conclusion that glycosylation of dCAT-1 is associated with MoMLV resistance.

**Table 3:** Virus titers of ecotropic gammaretroviruses on mouse cells and mouse or ferret MA139 cells transfected with dCAT-1-g

		**Log_10 _Virus Titer^a^**		
		
**Exp.**	**Cells^b^**	**F-S MLV**	**Spl574**	**MoMLV**
1	NIH 3T3	6.2	**2.5**	5.4
	*M. dunni*	6.0^c^	4.3^c^	**1.1**
	*M. dunni *(dCAT-1-g)	5.9^c^	4.5^c^	**2.6**

2	*M. dunni*	3.7^c^	4.1^c^	1.4
	MA139 (dCAT-1-g)	3.4^c^	**0.2**	3.9

^a^Virus titers were determined by the XC overlay test as in Table 1. Titers represent the number of XC PFU in 0.2 ml and were done in triplicate. **Bold** figures represent titers reduced by more than 95% (1.3 log_10_) relative to titers in susceptible NIH 3T3 or *M. dunni *cells.

^b^CAT-1 gene expressed in transfected cells is given in ().

^c^Syncytia were observed 2 days after virus infection.

MA139 cells expressing dCAT-1-g resembled FerrD2 in their susceptibility to virus infection (Table 3, Exp. 2 and Table 1) and sensitivity to F-S MLV-induced syncytia (Fig. 4B). The cells with the unglycosylated receptor were, like FerrD2, efficiently infected by MoMLV. Syncytia were produced in these transfectants with the same viruses that are cytopathic in FerrD2, and no cytopathic response was observed with viruses that are also noncytopathic in FerrD2. Thus, the complete absence of N-glycans on dCAT-1 did not alter the ability of the dCAT-1 receptor to mediate virus induced syncytium formation in MA139 cells, nor did it alter the panel of viruses that were cytopathic and/or infectious in the transfectants.

### Effect of glycosylation inhibitors on cellular proteins involved in virus entry

**Figure 5:**
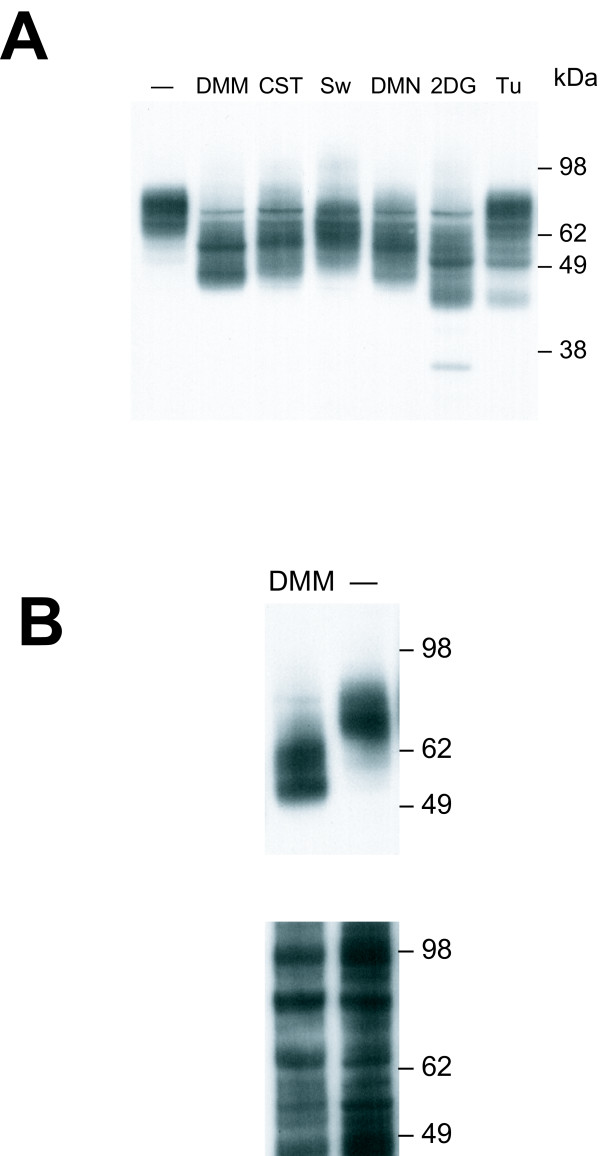
Effect of glycosylation inhibitors on expression of HA-tagged mCAT-1 in *M. dunni *cells. **(A) **Immunoblot analysis of lysates prepared from cells treated for 3 days with the indicated inhibitors: DMM, CST, Sw, DMN (65 μg/ml); 2DG (10 mM); Tu (0.125 ug/ml). **(B) **Immunoblot of surface biotinylated proteins from DMM-treated and untreated cells. The upper panel was probed with anti-HA; the lower panel shows the same blot stripped and reprobedwith streptavidin-HRP to show that surface biotinylation and protein loading were approximately equal.

The glycosylation inhibitor tunicamycin (Tu) was previously shown to reduce resistance to MoMLV in *M. dunni *cells [10]. We tested the ability of multiple glycosylation inhibitors to alter infectivity of ecotropic MLVs in mouse cells expressing the two functional CAT-1 variants: mCAT-1 (NIH 3T3 cells) and dCAT-1 (*M. dunni *cells). The 6 inhibitors included Tu which blocks generation of the carbohydrate-dolichol precursor needed for N-linked glycosylation, the sugar analog 2-deoxy-D-glucose (2DG), and 4 inhibitors which inhibit different enzymes involved in oligosaccharide trimming: castanospermine (CST), deoxymannojirimycin (DMM), deoxynojirimycin (DNM) and swainsonine (Sw). Western analysis of *M. dunni *cells transfected with HA-tagged mCAT-1 (Fig. 5A) showed that none of the inhibitors had a significant effect on expression levels, although all inhibitors reduced the size range of the mCAT-1 glycoprotein.

Because the resistance of NIH 3T3 cells to Spl574 infection is comparable to the resistance of *M. dunni *cells to MoMLV, we treated NIH 3T3 cells with 5 different glycosylation inhibitors before Spl574 infection (Table 4). All 5 inhibitors significantly reduced resistance to Spl574 replication, but inhibition of N-glycosylation did not affect the XC titer of other ecotropic viruses in NIH 3T3 cells, as shown for MoMLV. Resistance of SC-1 cells to Spl574 [1] is similarly relieved by glycosylation inhibitors (data not shown).

**Table 4:** Effect of various inhibitors of glycosylation on virus infectivity in *M. dunni *and NIH 3T3 cells

		**Log_10 _Virus Titer/Inhibitor^a^**					
		
**Virus**	**Cells**	**None**	**DMM**	**Sw**	**2DG**	**CST**	**Tu**
Spl574	NIH3T3	**3.1**	4.2	4.4	4.6	4.2	4.1
	*M. dunni*	5.7	5.8	NT	5.8	NT	5.8
MoMLV	NIH 3T3	5.7	NT	NT	5.9	NT	5.5
	*M. dunni*	**2.7**	4.4	4.7	4.9	4.1	4.5

^a^Virus was quantitated using the XC overlay assay [9] and numbers represent XC PFU in 0.2 ml. Glycosylation inhibitors were added the day before virus infection as follows: deoxymannojirimycin (DMM, 100 ug/ml); castanospermine (CST, 100 ug/ml), swainsonine (Sw, 200 ug/ml), 2-deoxy-D-glucose (2DG, 25 mM), tunicamycin (Tu, 0.2 ug/ml). NT, not tested.

*M. dunni *cells were also treated with the same set of glycosylation inhibitors prior to virus infection (Table 4). All inhibitors reduced the resistance of *M. dunni *cells to infection with MoMLV, but no comparable increase in titer was noted with Spl574. To confirm that this effect is on entry, DMM-treated *M. dunni *cells were infected with LacZ pseudotypes of MoMLV; pseudotype titer was 10^3.6 ^on DMM-treated cells compared to no detectable LacZ expressing cells in untreated *M. dunni*.

To determine if altered infectivity results from inhibitor-mediated changes in cell surface receptor levels, we measured biotinylated CAT-1 in *M. dunni *cells transfected with HA-tagged mCAT-1. As shown in Figure 5B, surface mCAT-1 in DMM-treated cells shows the expected reduction in size because of the predominance of smaller high-mannose N-glycans, but quantitation of this expression by densitometric scanning shows that the level in DMM treated cells is not significantly different from the untreated control.

These results, taken together, indicate that N-glycans can impede ecotropic MLV entry in cells expressing mCAT-1 as well as cells expressing dCAT-1, and that these N-glycans obstruct different ecotropic isolates in NIH 3T3 and *M. dunni *cells. Also, the fact that the effect on entry is seen with inhibitors other than Tu suggests that inhibition may be due to N-glycan type or size.

## Discussion

Three factors contribute to the observed variations in host range and/or cytopathicity of mouse ecotropic gammaretroviruses: specific sequence differences in the viral *env*, differences in the CAT-1 receptor, and glycosylation of cellular proteins. The role of specific *env *sequence variations in virus-induced syncytium formation was previously suggested by our identification of two MLV isolates that are uniquely cytopathic in *M. dunni *cells. Both isolates have amino acid substitutions at the same RBD residue that is critical for receptor binding: S82F in Spl574 and S84A in F-S MLV. That mutations in the viral receptor binding site contribute to cytopathicity is also supported by the observation that a third MLV variant, TR1.3, is cytopathic in SC-1 cells and brain endothelial cells because of a single substitution, W102G [13], at a site that together with S82 and D84 forms the receptor binding site [3,4].

The involvement of CAT-1 in the cytopathic response in *M. dunni *cells was suggested by the specific sequence differences that distinguish the dCAT-1 receptor variant from mCAT-1. These 2 receptors differ by 4 amino acids of which two are within the third extracellular CAT-1 loop that contains the virus binding site: I214V, and a glycine insertion within the YGE virus binding site [6]. As shown in the present paper, all cells expressing the dCAT-1 variant and none expressing mCAT-1 are susceptible to virus-induced syncytium formation. This indicates that one or both of these two amino acid changes, I214V and Δ236G, are responsible for the cytopathic response mediated by this receptor variant.

Previous studies with cytopathic retroviruses such as HIV have identified the accumulation of unintegrated DNA as a hallmark of cytopathicity [8]. Analysis of MA139 cells expressing the naturally occurring mouse receptor types, mCAT-1 and dCAT-1, shows that receptor type also correlates with this aspect of cytopathicity, and that in different dCAT-1 transfected lines the amount of unintegrated DNA corresponds to the extent of syncytium formation. This cell-virus system may thus be useful in further studies on the mechanisms thought to be involved in this cell killing such as endoplasmic reticulum stress induced apoptosis [14].

It is known that the glycans on various cell surface receptors can modulate virus entry (for example, [15]). The CAT-1 receptor is glycosylated at two sites, and previous studies have shown that glycosylation inhibitors reduce resistance to ecotropic MLV infection in rat and hamster cells expressing the rCAT-1 and haCAT-1 receptor variants [16-19], as well as resistance to MoMLV in *M. dunni *cells with dCAT-1 [10]. It has also been shown that in mink cells expressing mCAT-1, glycosylation affects SU binding and the down-modulation of receptor by virus infection [20]. Our results show that glycosylation modulates virus entry mediated by the laboratory mouse CAT-1 receptor, mCAT-1, in NIH 3T3 cells. This resistance is specific to Spl574 and is not seen in heterologous cells expressing mCAT-1. The control of this differential sensitivity of mCAT-1 to a specific ecotropic isolate by cell specific glycosylation has not been previously described.

The present study also considered whether altered glycosylation could explain why two cells expressing the same dCAT-1 receptor, *M. dunni *and FerrD2, produce syncytia in response to different viruses. As shown by the inhibitor results, however, while N-glycans contribute to the restriction of MoMLV entry into *M. dunni *cells, comparisons of ferret transfectants expressing the dCAT-1 or dCAT-1-g receptor variants produced no evidence that N-glycans modulate virus infectivity or virus-induced cytopathicity in the MA139 cells.

N-glycans can have high mannose, complex or hybrid structures. The various glycosylation inhibitors target different steps in protein glycosylation and can be used to manipulate the carbohydrate composition of glycoproteins. The inhibitor CST blocks glucose trimming, and DMM and SW inhibit successive steps in mannose trimming. The fact that all of these inhibitors along with the sugar analog 2DG and glycosylation inhibitor Tu relieved the resistance of *M. dunni *cells to MoMLV and of NIH 3T3 to Spl574 suggests that these viruses are most effectively blocked by the large complex oligosaccharides produced in the terminal stages of glycosylation. These results, taken together, suggest roles for N-glycans in virus entry that are virus-specific and cell-specific, and also indicate that this regulation may be sensitive to small sequence changes in both virus and receptor. These results indicate that N-glycans broadly regulate ecotropic gammaretrovirus interactions with the CAT-1 receptor in cells of their natural host [21], although it is possible that glycosylated proteins other than CAT-1 may contribute to this resistance.

Our demonstration that not all infectious viruses are cytopathic in *M. dunni *and FerrD2 cells supports the idea that virus-cell fusion and cell-cell fusion are distinct receptor-mediated phenomena. A similar lack of correlation between infectivity and syncytium formation has been reported, for example, in a mouse cell line that is unusual in its resistance to HTLV Env-mediated syncytium formation although it is highly susceptible to virus infection [22]. It has also been shown that, for a transformed NIH 3T3 cell line subject to MoMLV-induced syncytium formation, chloroquine treatment blocks MoMLV entry but does not also block syncytium formation [23]. Our results further distinguish cell fusion and virus entry as separate receptor functions.

Finally, these studies also identify differences between *M. dunni *and FerrD2 cells that are clearly not receptor mediated. Use of LacZ pseudotypes shows that Spl574 Envs efficiently mediate entry into FerrD2 cells, but XC titers in Spl574 virus infected FerrD2 cells are clearly reduced as is virus-induced syncytium formation. This indicates a post-entry block to virus replication leading to reduced surface Env, and the nature of this block is under investigation.

## Conclusion

The CAT1 receptor mediates ecotropic gammaretrovirus entry and the cytopathic response to virus infection. Use of virus *env *variants, receptor mutations, and inhibitors of glycosylation demonstrate that both of these virus-receptor interactions are modulated by a small number of critical amino acid residues in virus and receptor, and that N-linked glycans can modulate entry for specific virus-cell combinations.

## Methods

### Viruses

Three ecotropic MLV isolates were obtained from J. W. Hartley (NIAID, Bethesda, MD): Moloney MLV (MoMLV) and two FrMLV isolates, F-S MLV and FBLV. F-S MLV is an N-tropic FrMLV isolate. FBLV (NB-tropic FrMLV) is a biologically cloned virus originally provided by R. Risser (U. Wisc., Madison, Wisc.). Spl574 was isolated from a *M. spicilegus *mouse neonatally inoculated with MoMLV [1]. The Spl574 *env *differs from MoMLV at a single amino acid, S82F.

Virus stocks were made by collecting culture fluids from infected or transfected cells. These stocks were titered by the XC overlay test [9] following infection of NIH 3T3, SC-1 [24], or *M. dunni *[5] and cells transfected with CAT-1 receptor. Cells were plated at 1–2 × 10^5^cells/60 mm dish and infected with 0.2 ml of appropriate dilutions of virus stocks in the presence of polybrene (4 ug/ml; Aldrich, Milwaukee, WI). Cells were irradiated 4 days after virus infection with ultraviolet light from germicidal bulbs (30 sec at 60 ergs/mm^2^) to kill the cells but not the virus, and were then overlaid with 10^6 ^XC cells/plate. XC cells produce plaques containing syncytia in response to focal areas of virus infected cells. Plates were fixed and stained 3 days later and examined for plaques of syncytia.

### Syncytium formation and inhibitors of N-linked glycosylation

To screen for the formation of multinucleated syncytium in virus infected cells, 2 × 10^4 ^cells in six-well tissue culture plates or 10^5 ^cells in 60 mm plates were infected with virus-containing medium in the presence of 4 ug/ml polybrene. After 2–4 days, the cells were examined by light microscopy using objective lenses of 4×–20× and photographed using a Nikon TS100 microscope and digital camera DXM1200.

Cells were treated prior to virus infection by various inhibitors of N-linked glycosylation: deoxymannojirimycin (DMM); castanospermine (CST), swainsonine (Sw), deoxynojirimycin (DMN), 2-deoxy-D-glucose (2DG) and tunicamycin (Tu). All inhibitors were obtained from SIGMA (La Jolla, Calif.) Inhibitors were added to cultures that had been seeded the previous day; virus was added the next day and inhibitors and polybrene were removed the following day for the XC plaque assay, but were not removed prior to lysis of the cells for immunoblotting.

### Cloning and mutagenesis

The CAT-1 receptor variant of *M. dunni*, dCAT-1, was amplified from *M. dunni *cells by RT-PCR with forward (mdCAT1: CTGTGCTACGGCGAGTTTG) and reverse (mdCAT2: TCCACCAGGTCCTTCAGTTC) primers derived from the NIH 3T3 ecotropic receptor sequence (GenBank accession no. M26687). The 965 bp product was cloned into the pCR2.1-TOPO vector (Invitrogen Co., Carlsbad, Calif.) and sequenced. The deduced amino acid sequence of the third extracellular loop (Fig. 1) was identical to that described by Eiden and her coworkers [6]. The HpaI-Bsu36I fragment of this dCAT-1 receptor fragment was used to replace the corresponding fragment of plasmid pcdna3:MCAT-1Flutag which contains the HA-tagged NIH 3T3 CAT-1 receptor (mCAT-1) and was a gift of J. M. Cunningham (Harvard Medical School, Boston, MA). This fragment contains 3 of the 4 amino acid differences that distinguish dCAT-1: I214V, Δ236G, and N373D. N373D lies in the 4th putative intracellular loop.

Mutations were introduced into both potential N-glycosylation sites of dCAT-1 by a PCR-based protocol. The substitutions N223E and N229V were introduced because mCAT-1 with these mutations is functional [20]. The sense primer was 5'-CTCACGGAGAAAGAATTCTCCTGTAACAACGTCGACACAAACG-3' (G1 primer) and the antisense primer was 5'-CGTTTGTGTCGACGTTGTTACAGGAGAATTCTTTCTCCGTGAG-3' (G2 primer). PCR reactions used the dCAT-1 construct as template. In the first reaction, the sense primer G1 was used with the antisense primer 5'-TGAAACCTATCAGCATCCACACTG-3' from the 3' end of the CAT-1 gene (GenBank Accession No. M26687). In the second reaction, the antisense primer G2 was used with the sense primer 5'GCGGATCCTAATGGGCTGCAAAAACC-3' from the 5' end of the CAT-1 gene. The combined products of these two reactions provided the template for an additional PCR using the flanking 5' and 3' primers. The amplified product was digested with AgeI and FseI to generate a 1.1 kb fragment containing the mutant sequence. This fragment was ligated into the dCAT-1 clone to generate dCAT-1-g. The presence of the two mutations was confirmed by restriction digestion to identify the novel SalI and EcoRI sites at the mutation sites, and by sequencing (Fig. 1).

### Generation of transfected cells and analysis for unintegrated DNA

DNA clones of mCAT-1, dCAT-1 and dCAT-1-g were introduced into cultured cells using the FuGENE 6 transfection reagent (Roche Applied Sci., Indianapolis, IN). Cells used for transfection included *M. dunni *cells [5], MDCK (canine kidney, ATCC-CCL 34), Tb-1-Lu (bat lung, ATCC-CCL 88) and MA139 (ferret, obtained from J. Hartley). Cells were trypsinized and passed 24 hours after transfection and maintained in medium with 0.8 mg/ml geneticin (Invitrogen, Grand Island, NY) until colonies of drug resistant cells were apparent. Individual colonies were picked as indicated or were pooled for analysis.

Unintegrated viral DNA was extracted from virus infected cells by the Hirt method [25]. These DNAs were digested with EcoRI, separated on agarose gels and hybridized with a 306 bp segment of the ecotropic *pol *gene as described previously [2].

### Pseudotype assay

LacZ pseudotype virus was generated by cotransfection of human 293 cells with pCLMFG-LacZ (Imgenex Co., San Diego, Calif.) and expression vectors containing various ecotropic MLV *env *genes. The pCL-eco retrovirus packaging vector (Imgenex Co., San Diego, Calif.) was used to generate pseudotypes with Moloney ecotropic Env. Substitutions in this vector were used to generate pseudotypes containing the 5'*env *of FrMLV57 and Spl574 as described previously [1,2].

Supernatants containing pseudotype virus were collected from transfected human 293 cells, filtered and used to infect cells that had been plated in 12-well culture dishes. The cells were infected with appropriate dilutions of pseudotype virus in the presence of 4–8 μg/ml polybrene. One day after infection, cells were fixed with 0.4% glutaraldehyde and assayed for β-galactosidase activity using as substrate 5-bromo-4-chloro-3-indolyl-β-D-galactopyranoside (X-Gal, 2 mg/ml; ICN Biomedicals, Aurora, Ohio). Infectious titers were expressed as the number of blue cells per 100 microliters of virus supernatant.

### Western immunoblotting

Transfected cells were tested for expression of HA-tagged CAT-1 by Western immunoblot analysis. Cell lysates were subjected to electrophoresis on NuPAGE 4–12% Bis-Tris Gels (Invitrogen) or on sodium dodecyl sulfate polyacrylamide gels (8% or 10%). Subsequent immunoblot analysis used a mouse anti-HA monoclonal antibody (clone 12CA5) and peroxidase conjugated goat anti-mouse IgG (gamma 2b) (Roche Applied Sci., Indianapolis, IN).

Surface proteins were biotinylated using a membrane-impermeant biotin reagent from Pierce (catalog no. 21327; Rockford, IL). Proteins were purified using streptavidin beads (Pierce, catalog no. 29200) and analysed by Western blotting using anti-HA antibody. The membrane was stripped using Pierce Stripping Buffer (catalog no. 21059) and reprobed using HRP-conjugated streptavidin (Pierce; catalog no. 21126).

## Competing interests

The author(s) declare that they have no competing interests.

## Authors' contributions

YTJ and YY made the dCAT-1 and Env constructs and did the pseudotype experiments. YY did the unintegrated DNA analysis. YY and TW did the Western analysis and virus infections. CK did the cytopathicity tests and drafted the manuscript. All authors read and approved the final manuscript.
